# Effect of feeding toy and the presence of a dog owner during the feeding time on dog welfare

**DOI:** 10.14202/vetworld.2023.1721-1726

**Published:** 2023-08-24

**Authors:** Worakan Boonhoh, Tuempong Wongtawan, Prarom Sriphavatsarakom, Natalie Waran, Phatcharaporn Chiawwit, Noppharat Tanthanathipchai, Naparat Suttidate

**Affiliations:** 1 College of Graduate Studies, Walailak University, Nakhon Si Thammarat, 80160, Thailand; 2 Akkhraratchakumari Veterinary College, Walailak University, Nakhon Si Thammarat, 80160, Thailand; 3 Center of Excellence in Innovation on Essential Oils and Bioactive Compounds, Walailak University, Nakhon Si Thammarat, 80160, Thailand; 4 One Health Research Center, Walailak University, Nakhon Si Thammarat, 80160, Thailand; 5 Department of Preclinic and Applied Animal Science, Faculty of Veterinary Science, Mahidol University, Nakhon Pathom, 73170, Thailand; 6 Faculty of Education, Humanities and Health Science, Eastern Institute of Technology, Hawke’s Bay, 4142, New Zealand

**Keywords:** cortisol, dog behavior, dog welfare, dog-human relationship, feeding toy, heart rate variability

## Abstract

**Background and Aim:**

A conventional feeding bowl is the primary method that dog owners use to feed their dogs, but this may not encourage natural behaviors and may even exacerbate unwanted behaviors. This study aimed to compare a conventional feeding bowl to a feeding toy in relation to behavior, cortisol levels, and heart rate variability (HRV).

**Materials and Methods:**

The behaviors of four dogs were recorded and analyzed while being fed using either a stainless bowl (B) or a feeding toy (T) and either alone (A) or accompanied by a dog owner (O) for 30 min with each treatment (BA, BO, TA, and TO treatments). The dogs that were fed alone with the stainless bowl (B_C_) or the feeding toy (T_C_) were fed for 15 min/day for 7 days with their treatment, and serum cortisol levels measured on the first and last days of treatment. The dogs fed by the stainless bowl (B_H_) or the feeding toy (T_H_) with the owner present for 15 min for each treatment had their heart rate (HR) and HRV recorded by Polar^®^ H10 during feedings The results were compared using a one-way analysis of variance (ANOVA), repeated measure ANOVA, and Student’s t-test.

**Results:**

The dogs spent more time eating and interacting with the feeding toys than stainless bowls. The activity of the dogs was higher when using feeding toys, particularly with the TO treatment. Cortisol levels were significantly lower on day 7 than on day 1 of the T_C_ treatment. The dogs’ HR was higher during T_H_ treatment than during B_H_ treatment. All HRV parameters were decreased significantly when feeding the dog with the toys.

**Conclusion:**

The results of this study support the idea that feeding enrichment supports the natural feeding behaviors of dogs as they mimic hunting and playing behaviors. This reduced unwanted behavior, cortisol levels, and HRV, and increased food consumption, eating duration, and active behaviors. The presence of the dog’s owner is important because it can enhance feeding and active behaviors, and feeding enrichment can improve the dog’s welfare and the dog-human relationship.

## Introduction

Dogs are the world’s most popular pets and play an important role in human life. Dogs and pet ownership can improve physical and mental health [1, 2]. Owning a pet may help reduce the risk of heart disease because dog owners engage in more physical activity, including walking and running than others [3–5]. However, canine behavior problems such as separation anxiety, aggression, and fearfulness could disturb human well-being. Separation-related behavior occurs in 14%–20% of dogs [6], including destructive behaviors, vocalization, and inappropriate elimination. Dogs with separation anxiety had cortisol levels twice that of normal dogs [7], and cortisol is a well-known stress marker in dogs [8]. Cortisol is normally released in response to stress. When cortisol is overproduced, it can take up to 72 h for it to be metabolized. This may result in canine misbehavior during elevated levels [9].

There are multiple signs of stress in dogs, including whining, barking, yawning, drooling, licking, shedding, panting, hiding, pacing, shaking, and displacement behaviors. The best way to relieve stress in dogs is to avoid stressful situations. Physical exercise, walking, or playing can greatly reduce stress [10].

Feeding behavior may be related to behavior problems. The primary method of feeding uses a stainless bowl. This conventional feeding technique is boring [11], does not sufficiently mimic natural behavior, and may be related to obesity [12, 13]. In free-roaming dogs, food sources such as carcasses require considerable eating time, approximately 26 min [14]. In addition, feeding toys are also preferred by dogs [15].

Feeding enrichment, such as feeding toys, may improve natural hunting behavior, reduce unwanted behaviors, and keep dogs mentally engaged and physically indulged. Heart rate variability (HRV) is used to study sympathovagal activity related to physical and mental responses. Heart rate variability is a non-invasive technique for autonomic nervous system measurement, which plays an important role in monitoring the stress response by measuring fluctuations in the RR interval (RRI). There is increasing use of HRV in veterinary research studies and clinical practice worldwide, including in Thailand [16–18]. The duration of heart rate (HR) or electrocardiogram recordings using time-domain or frequency-domain measurements can vary from <1 min to more than 24 h [19]. Previous studies by Bidoli *et al*. [20] found that cardiac monitoring with HRV was consistent with the dog’s behavior, and parasympathetic deactivation was associated with a more positive emotional state and autonomic effects related to postural shifts [21]. Polar® human HR monitor equipment is usually used in HRV research on dogs [22, 23]. The Polar device has an artifact rate of ≤5%, which makes it suitable for analysis of HR and HRV monitoring [20].

This study aimed to evaluate a conventional feeding method using a feeding toy as it related to dog behavior, cortisol levels, and HRV. This study also aimed to determine whether this could enhance feeding and active behaviors in dogs and improve dog welfare or dog-human relationships.

## Materials and Methods

### Ethical approval

This study was approved by the Institutional Animal Care and Use Committee of Walailak University (WU-ACUC-65004).

### Study period and location

The study was conducted from January to May 2022 at the Animal Laboratory Unit, Small Animal Teaching Hospital, Akkraratchakumari Veterinary College, Walailak University, Thailand.

### Animals

Four healthy, mixed breed dogs were studied, aged 3.25 ± 0.50 years old, weighing 13.5 ± 3.11 kg, from a dog shelter in Nakhon Si Thammarat, Thailand. They all received annual vaccinations (distemper, adenovirus type 2, parainfluenza, parvovirus, leptospira, coronavirus, and rabies) and were injected with ivermectin regularly for parasite prevention. The dogs underwent physical and blood examinations, including complete blood count, alanine transaminase, creatinine, and blood urea nitrogen, performed by a veterinarian for a routine health check. Blood parasite test kit screening (SNAP 4Dx Plus test, IDEXX, USA) was also performed since the dogs were from a shelter at risk of blood parasite infection [24, 25]. They were treated and quarantined at the animal laboratory unit, Small Animal Teaching Hospital, Akkraratchakumari Veterinary College, for 30 days to become familiar with and recognize the researcher (WB) as their owner before starting the experiments.

### Dog behavior experiments

**Figure-1:**
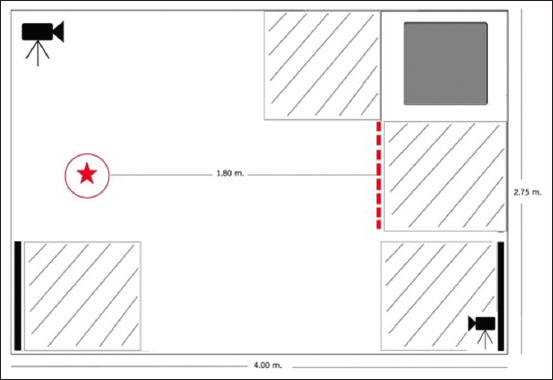
The dog behavior experimental room layout in the study; room size 4.00 × 2.75 m^2^, solid blacks object with tripods represents camera placement in the room, the circle represents the placement of a dog toy or a dog bowl at the star inside, the dashed line represents starting line of dogs which the length between the dashed line and the circle is 1.80 m, the solid gray square shape represents a chair where the researcher (WB) sit, the white 90 × 90 cm squares with slash lines represent dog positioning close to the chair and close to the doors, and the black solid lines represent the sliding doors.

All experiments were conducted in the 4.00 × 2.75 m^2^ room (Figure-1). Two video cameras on tripods (GoPro Hero 9 Black, GoPro, Inc., USA) were used to record the dogs’ behaviors. A plastic, bowling pin-shaped, and gravity ball-based feeding toy was used to represent feeding enrichment and was compared with a conventional stainless feeding bowl. Eighty grams of pellet food were used in each experimental treatment. Each treatment consisted of one of the feeding methods, and another 120 g of food was given to the dogs afterward in feeding bowls in their cages.

There were three experiments in this study. In the first experiment, in the experimental room, dogs were introduced to toys and bowls for 30 min a day for 3 days (pre-toy period). Experiment 1 consisted of feeding the dogs with either a feeding bowl (B) or a feeding toy (T) and either alone (A) or with the owner (O), keeping them company but without any interaction. This resulted in four treatment options: BA, BO, TA, and TO. Each option was performed and recorded by video for 30 min once daily. The next treatment option was performed on the next day in a randomized order. The dog behavior was analyzed using the Solomon coder program version Beta 19.08.02 (https://solomon.andraspeter.com/) with the ethogram, as shown in Table-1.

**Table-1:** Ethogram of dog behaviors in experiment 1 of the present study.

Behaviors	Description
First approach	Time (seconds) from the starting line to the dog entering the object area marked on the ground
Eating duration	The duration (seconds) starts from the dog putting its mouth into the bowl/toy picking up the kibbles and stopping when the dog ceases chewing or moves away from the bowl/toy.
Frequency approach	The number of times the dog intentionally touches the object by either mouth, nose, or paw, OR the dog intentionally moves his nose toward the object when the object is positioned less than half the length of the dog’s body.
Toy interaction	The duration (seconds) of the dog playing with the feeding toy includes;
	• Right pawing: Time from when the dog moves their right front paw toward the toy to when the right front paw is back on the ground.
	• Left pawing: Time from when the dog moves their left front paw toward the toy to when the left front paw is back on the ground.
	• Nosing and sniffing: Time the dog’s nose touches OR the nose is intentionally moved toward the toy.
	• Mouthing: Time the toy is in contact with the dog’s mouth/teeth.
	• Vocalizing: Time the dog emits voice toward the toy with the body oriented to the toy.
Passive behaviors	The duration (seconds) of the dog doing these passive behaviors including lying down quietly, sitting quietly, sleeping, standing, and walking
Vocalization	The duration (seconds) starts from the dog vocalizes but not at the object including howling, barking, yelping, and whining
Destructive behaviors	The duration (seconds) of the dog doing these destructive behaviors including door scratching, wall scratching, and floor scratching
Displacement behavior	The duration (seconds) of the dog doing these displacement behaviors including dry shaking, yawning, stretching, scratching, and grooming
Stay close to the chair	The duration (seconds) of the dog stay close to the chair as the layout described in Figure-1
Stay close to the doors	The duration (seconds) of the dog stay close to the doors as the layout described in Figure-1

In the second experiment, dogs were fed in the experimental room for 15 min once a day for 7 consecutive days for each treatment option in a randomized order. The two options were conventional bowl treatment (B_C_) and feeding toy treatment (T_C_). There was no owner present in experiment 2. Blood was collected from the dogs at the beginning of each option on day 1 and immediately after their 15 min of treatment on day 7. Serum cortisol levels were measured using the enzyme-linked immunosorbent assay technique as previously described by Brown *et al*. [26].

In the third experiment, the dogs’ HR and HRV were measured using the Polar® H10 HR monitor (Polar® H10, POLAR, Finland) during feeding for 15 min each day during the experimental procedures. There were two treatments in experiment 3: The bowl (B_H_) and the feeding toy (T_H_). Dogs were with the owner (WB) during both feeding options; however, the owner was only there to keep the dog company and to observe the equipment. The owner had no interaction with the dogs. The dogs’ hair was clipped along the chest, ultrasound gel was applied to the area, and the Polar^®^ H10 was strapped on. An elastic bandage was applied to cover the chest strap for stability when the dog moved. The Polar^®^ H10 was connected through Bluetooth to Polar Beat: Running and Fitness and HR and HRV Logger 1.3.5 applications on the smartphone (Android system).

### Statistical analysis

The data were analyzed using Jamovi 2.3.9 software (The Jamovi project, Sydney, Australia) for statistical analysis and presented as mean ± standard deviation. One-way analysis of variance (ANOVA) and Tukey’s *post hoc* test were performed to analyze data among the four treatment options in experiment 1. Repeated measurement of ANOVA followed by Tukey’s *post hoc* test was used to analyze cortisol levels in experiment 2. HR and HRV parameters between the bowl and toy treatments in experiment 3 were analyzed by student’s t-test. p < 0.05 was considered statistically significant.

## Results

### Feeding strategy effects on dog behavior

The dogs consumed significantly more food in the BO treatment (p = 0.023) compared with the BA treatment; however, consumption was not different between the BO and TA or TO treatments (p = 0.135 and p = 0.942, respectively). The first approach time to the bowl or toy was not significantly different among the BA, BO, TA, and TO treatments. The dogs spent the most time eating in the TO experiment, and it was significantly comparable with the BA and BO treatments (p = 0.007 and p = 0.020, respectively). It was similar to the frequency approach the object results (p = 0.035 and p = 0.029, respectively). Toy interaction duration was not significantly different between the TA and TO treatments (p = 0.113). Two of the dogs in the study seemed to be right-pawed, one seemed to be left-pawed, and the other used both of its forelimbs to play with the toys. Nosing and sniffing were the longest interaction times in the TA and TO treatments (95.50 ± 94.80 and 126.00 ± 31.90 s, respectively), while mouthing was the shortest duration of toy interaction (9.15 ± 17.90 and 3.85±7.70 s, respectively). Half of the dogs did not use their mouths to interact with the toys (Table-2).

**Table-2:** Feeding strategy effects on food consumption and dog behavior (Mean ± SD).

Parameter	Type	Unit	BA	BO	TA	TO
Food consumption	Volume	Grams	35.80^a^ ± 30.92	80.00^b^ ± 0.00	49.30^a,b^ ± 18.93	72.80^a,b^ ± 4.79
First approaching	Duration	Seconds	5.25 ± 4.34	2.10 ± 1.04	2.10 ± 0.87	3.75 ± 2.72
Eating duration	Duration	Seconds	102.00^a^ ± 108	161.00^a,b^ ± 112.00	272^a,b,c^ ± 161.00	492.00^c^ ± 147
Frequency approaching	Frequency	Times/session	6.75^a^ ± 2.99	6.25^a,b^ ± 1.71	18.25^a,b,c^ ± 8.42	22.50^c^ ± 10.66
Toy interaction	Duration	Seconds	NA	NA	161.00 ± 66.80	337.00 ± 187.60
Passive behavior	Duration	Seconds	1593.00^a^ ± 161	1519.00^a,b^ ± 130.00	1378.00^a,b,c^ ± 202.00	1045.00^c^ ± 270.00
Vocalizing	Duration	Seconds	27.65 ± 23.67	10.40 ± 15.00	43.55 ± 35.84	5.35 ± 4.77
Destructive behavior	Duration	Seconds	38.45 ± 68.38	3.65 ± 7.30	15.90 ± 16.51	13.65 ± 20.52
Displacement behavior	Duration	Seconds	36.70 ± 17.50	41.20 ± 16.00	45.30 ± 17.90	85.80 ± 93.30
Stay close to the chair	Duration	Seconds	56.40^a^ ± 55.10	1096.00^b^ ± 339.50	257.00^a^ ± 312.60	439.10^a^ ± 333.30
Stay close to the doors	Duration	Seconds	594.50 ± 684.10	82.70 ± 42.10	289.90 ± 318.60	84.70 ± 92.30

Abbreviations: ^a,b,c^Different letters represent significant differences (p < 0.05), BA=Feeding bowl alone treatment, BO=Feeding bowl with owner treatment, TA=Feeding toy alone treatment, TO=Feeding toy with owner treatment

The dogs’ passive behavior was the longest in the BA treatment option. Both feeding bowl treatments, BA and BO, had passive behavior duration significantly longer than the TO treatment (p = 0.010 and p = 0.024, respectively). There was no significant difference in vocalizing, destructive behavior, or displacement behavior among the BA, BO, TA, and TO treatments in this study. In the BO treatment, the dogs spent 60% of the session close to the chairs where the owner sat, and it was significantly longer compared to the other treatments (Table-2).

### Feeding strategy effects on serum cortisol levels

Serum cortisol levels in the B_C_ treatment option showed no difference from day 1 to day 7, (p = 0.103) while the T_C_ group exhibited a significant decrease between day 1 and day 7 (p = 0.025) with 2.28 (ng/mL) mean difference (Table-3).

**Table-3:** Feeding strategy effects on serum cortisol levels of the dogs.

Treatments	Serum cortisol levels (ng/mL)		

	Day 1 (Mean ± SD)	Day 7 (Mean ± SD)	Mean difference
B_C_	6.31 ± 0.58	4.32 ± 0.51	1.99
T_C_	5.96^a^ ± 0.10	3.68^b^ ± 0.84	2.28

^a,b^Different letters represent significant differences (p < 0.05), B_C_=Feeding bowl treatment in the cortisol experiment, T_C_=Feeding toy treatment in the cortisol experiment

### Feeding strategy effects on HR and HRV

The average HR was significantly higher during the T_H_ treatment option than the B_H_ treatment (p = 0.035). All HRV parameters in the B_H_ treatment, including average RRI, standard deviation of normal sinus beats (SDNN), root mean square difference (RMSSD), and the proportion of RRI exceeding 50 ms (PNN50) were significantly higher than in the T_H_ treatment option (p = 0.031, p = 0.021, p = 0.030, and p = 0.016, respectively) (Table-4).

**Table-4:** Feeding strategy effects on heart rate and heart rate variability.

HRV parameter	Unit	B_H_ (Mean ± SD)	T_H_ (Mean ± SD)
HR	BPM	96.50^a^ ± 13.48	106.00^b^ ± 15.64
RRI	ms	675.50^a^ ± 92.97	585.50^b^ ± 80.66
SDNN	ms	165.50^a^ ± 47.19	121.00^b^ ± 42.17
RMSSD	ms	144.50^a^ ± 44.13	100.75^b^ ± 35.17
PNN50	%	43.25^a^ ± 11.64	26.50^b^ ± 12.40

^a,b^Different letters represent significant differences (p < 0.05), HR=Heart rate, HRV=Heart rate variability, BPM=Beat per minute, RRI=RR interval, SDNN=The standard deviation of normal sinus beats, RMSSD=The root mean square of successive differences between normal heartbeats, PNN50=The percentage of adjacent NN intervals that differ from each other by more than 50 ms, B_H_=Feeding bowl treatment in the HRV experiment, T_H_=Feeding toy treatment in the HRV experiment

## Discussion

In this study, dogs tended to consume more food when feeding with the bowl than using feeding toys, whereas they tended to spend more time with the toys than with the bowls. Dogs spent only 2–3 min when eating with the bowl, but spent 5–10 min when eating with a feeding toy. They likely would have spent more time with the feeding toy if given more than 80 g of feed. The feeding toy elicited more interest from the dogs than the bowl, especially when the owner accompanied them. These results are similar to those of previous studies, which found that dogs preferred feeding toys [15], and the toys stimulated appetitive behaviors, increased exercise activity, and lowered barking frequencies [27]. The human owner may influence dogs to play and interact with the toy more than when they are left alone with the toys. This could be due to positive reinforcement related to attention to the owner when the dogs play with the toys. The dogs had less passive behavior with the feeding toys than when fed with the bowls. These results may be related to improved natural hunting behavior using the feeding toy instead of the conventional stainless bowl.

Domestic dog paw preference reflects the dominant limb or hemispheric functioning of the brain, which may be influenced by sex [28]. Two female dogs in the present study were right-paw biased based on their interaction with the toys. One male was left-paw biased, and the other female used both paws. Female dogs are more likely to be right-paw biased, while males are more prone to be left-pawed [28], similar to this study’s findings.

Dogs respond to long-term stress with either behavioral or hormonal changes, including body shaking, yawning, stretching, vocalizing, other displacement behaviors, destructive behavior, and increasing cortisol, adrenaline, and noradrenaline levels [29]. Reducing stress in dogs could be achieved with environmental enrichment such as toys. Feeding toys could decrease unwanted behaviors and increase natural or instinctive behaviors in dogs [12]. This is similar to the results of this study, where the serum cortisol level decreased after the dogs were fed with feeding toys for 7 days. In addition, displacement and destructive behavior decreased, especially when the owner accompanied them. Free-roaming dogs chewed on food for approximately 26 min, which fulfilled the dog’s instinct and also improved their oral cavity health by reducing plaque and tartar [14]. The feeding toy in this study increased the dog’s eating time which may have fulfilled their feeding instinct, reduced stress, and improved their welfare.

When the dogs played and ate with the toys, HR increased but HRV decreased. The increased HR was associated with more physical activity using their paws, nose, and mouth during toy interaction. A previous study by Wells [30] found that dog toys encouraged exploration and decreased habituation by allowing dogs to spend more time in moving and less time in standing, which is similar to our results. Previous studies by Shaffer and Ginsberg [19], and Gevirtz *et al*. [31] using toy treatment found that decreasing HRV may be related to respiratory regulatory mechanisms that control HR, with an increase in respiratory rate and a rise in sympathetic nervous system activity suppressing parasympathetic nervous system activity. This study found that all HRV parameters, including SDNN, RMSSD, and PNN50, were significantly lower when dogs were fed with toys. This is related to a positive emotional state and a changing body position, as noted by Zupan *et al*. [21].

## Conclusion

Feeding enrichment may stimulate natural hunting and feeding behaviors by reducing unwanted behaviors, cortisol levels, and HRV and increasing active behaviors. This is beneficial for both the physical and mental health of dogs. The dog owner is an important factor who can positively impact feeding consumption, eating duration, and active behaviors. A feeding enrichment strategy can help improve dog welfare and the dog-human relationship.

## Authors’ Contributions

TW, NS, PS, and WB: Designed the study, prepared the materials, and edited the manuscript. WB: Performed animal experiments, coded dog behavior, collected and analyzed the data, and drafted and revised the manuscript. NW: Commented and advised on the experiment and the manuscript. PC and NT: Helped on data collection and analysis. All authors have read, reviewed, and approved the final manuscript.
